# Drug-Resistant Tuberculosis Stigma Among HealthCare Workers Toward the Development of a Stigma-Reduction Strategy: A Scoping Review

**DOI:** 10.1177/00469580231180754

**Published:** 2023-06-13

**Authors:** Lolita Liboon Aranas, Khorshed Alam, Prajwal Gyawali, Rashidul Mahumud Alam

**Affiliations:** 1 University of Southern Queensland, Toowoomba, QLD, Australia; 2 Jose Rizal University, Mandaluyong City, Philippines; 3 The University of Sydney, Sydney, Australia

**Keywords:** drug-resistant tuberculosis, DRTB stigma, health workers, stigma, TB stigma, tuberculosis stigma

## Abstract

Drug-resistant tuberculosis (DRTB) is a growing concern worldwide. The poor rate of service delivery exacerbates the severity, leading to an increase in community transmission, which is further amplified by stigma. Health care workers (HCWs) are at the forefront lines of service delivery; their efforts are suspected of resulting in stigmatization, negatively impacting patient-centered care. However, little is known about DRTB-related stigma among these HCWs, and interventions are limited. Our scoping review is significant because it provides an overview of the DRTB stigma confronting HCWs and informs subsequent stigma-reduction initiatives. Utilizing Arksey and O’Malley framework, we exhaustively searched electronic databases for relevant English-language studies published from 2010 to 2022, identifying the drivers and facilitators of DRTB-related stigma among HCWs from high-TB and -DRTB burden countries, and compiling recommendations that could reduce DRTB stigma. From 443 de-duplicated papers, 11 articles on HCWs’ DRTB-related stigma were reviewed and synthesized. Fear was mentioned across included articles as a stigma driver. Other reported stigma drivers identified included feelings of discrimination, isolation, danger, lack of support, shame, and stress. Poor infection control (IC) was the leading stigma facilitator. Other stigma facilitators identified were differing IC interpretations, workforce culture, and workplace inequality facilitating to stigmatization of HCWs. Three key recommendations identified were addressing infection control issues; increase the competence of healthcare workers; and provide psychosocial assistance, emphasizing HCW safety during DRTB activities. DRTB stigma among HCWs is multifaceted, largely driven by fear and facilitated by varying implementation or interpretations of policies within the workplace. Making HCWs feel safe while conducting DRTB activities is a priority issue that should be addressed by improving IC, training and psychosocial support. More studies investigating country-specific and multilevel DRTB-related stigma among HCWs are required to inform the development of an effective stigma intervention strategy.

**What do we know so far about this topic?**
Health workers are on the front lines of DRTB service delivery; their efforts in all areas of disease control are suspected of resulting in stigmatization, which has a negative impact on patient-centered care.
**What contribution does this research make to the field?**
This is the first study to provide an overview of the DRTB stigma drivers and facilitators, which could assist program managers and policymakers in identifying the policies that need to be addressed to create a stigma-free healthcare environment.
**What are the implications of the research for theory, practise, or policy?**
Policymakers must examine TB and DRTB policies, particularly IC and risk-payments, in order to minimize disparities and develop viable, effective solutions that discourage DRTB factors that driver and facilitator stigma among HCWs.

## Introduction

Tuberculosis (TB) is a global public health concern.^1^ About a fourth of the world’s population had latent TB in 2020, and 10 million had active TB.^1^ Alarmingly, TB drug resistance is growing and affecting about 500 000 people annually, and its persisting increase is an urgent and challenging obstacle to TB control and prevention.^2^

Combatting DRTB requires therapeutic efficacy, equity, and safety, as well as patient-centered care.^3^ Patient-centered care addresses socioeconomic issues that worsen DRTB, like poverty and geographic barriers to health care. Its holistic approach rewards patients, treatment advocates, and healthcare providers, minimizing stigma.^4^ Healthcare workers (HCWs) involved in DRTB activities and psychosocial support are integral to patient-centered care.^5^

TB is a stigmatized disease.^6^ Stigma is the community’s labeling of a trait as undesirable or devalued, which can lead to disgust, fear, guilt, and shame.^7,8^ In healthcare, TB-related stigma is commonly associated with “dirty work,” and HCWs delivering care for TB are viewed as facing the dirty work stigma.^9^

The stigma of TB causes diagnostic delays and treatment refusals. Stigmatized TB patients are reluctant to seek and complete treatment^10^; whereas stigmatized HCWs exhibit undesirable behaviors toward their patients or co-workers.^11^ However, stigma’s impacts extend beyond disease prevention and treatment, affecting the quality of life of patients and those around them, including HCWs^12-15^; consequently, DRTB spread.^2^

DRTB stigma is pronounced,^2^ and patients are particularly vulnerable to it.^16^ The psychosocial concerns surrounding the disease disrupt the social lives of patients and their families.^17-19^ Similarly, stigma in DRTB affects HCWs’ well-being and often leads to stigmatizing behaviors in healthcare settings,^11^ thus, a major barrier to patient-centered care.^20-22^

Stigmatization mechanisms frequently share characteristics, but their consequences can differ.^11^ For instance, many DRTB patients experience depression, guilt, loss of self-identity or self-esteem^23^; while others experience isolation, relationship failure, or separation.^24^ Despite a lot of study on the consequences of DRTB stigma on patients and their families, no study has systematically examined the stigma confronted by HCWs.

As a growing concern, reducing DRTB workplace stigma is important^11^ and calls for a sustainable response that should be addressed at all levels.^9,25^ At the facility level, targeting the factors comprising the stigmatization process is increasingly recognized. Stigma factors may include illness characteristics (eg, virulence), features from within the facility (eg, policies), and individual HCW (eg, attitude).^25^ However, measures addressing workplace health stigma are lacking.^26^ Thus, this review on DRTB-associated stigma among HCWs is significant to TB program managers and researchers to build sustainable stigma reduction efforts.

## Methods

Part of a larger study on DRTB stigmatizing Filipino HCWs, this review highlights materials that may influence future research and decision-making.^27,28^ Unlike systematic review, which synthesizes primary studies to address a specific topic to minimize bias,^29^ this review combines material and finds gaps in workplace DRTB-related stigma.^28^

This review aimed to (1) acquire a better understanding of the stigma surrounding DRTB; and (2) provide insights that could inform the development of interventions to reduce DRTB stigma among HCWs. DRTB-related stigma factors affecting HCWs were identified by identifying stigma drivers and facilitators and HCW stigma experiences. We collected DRTB stigma-reduction recommendations.

This review utilized Arksey and O’Malley framework,^30^ Peters et al’s recommendations^31^ and the Joanna Briggs Institute System for the Unified Management of the Assessment and Review of Information (JBI SUMARI) utilities. To ensure systematic reporting, the PRISMA Extension for Scoping Reviews (PRISMA-ScR) checklist was used.^32^ Prior to conducting this review, this scoping review was registered in Open Science Framework osf.io/43kp9 and a protocol was published.^33^

### Study Selection

Preliminary search was conducted in February 2022 and updated in September–November 2022. Peters et al’s^34^ Population, Concept, and Context (PCC) criteria (Table 1) guided our search strategy, focusing on 2 concepts – DRTB and stigma. The search followed JBI scoping review protocol—pilot search, search protocol preparation, and database search utilizing Medical Subject Heading (MeSH) terms (Supplemental Appendix 1).

**Table 1.** Scope of Inquiry for Examining DRTB Stigma Drivers and Facilitators Among HCWs.

Participants	*Health workers—*including physicians, nurses, midwives, medical technologist, pharmacists, and other allied professionals in healthcare settings such as hospitals, clinics, community centers, and TB treatment facilities delivering DRTB services such as case-finding, screening, diagnosis, treatment, and prevention.
Concept	*Stigma of health workers delivering services to DRTB patients -* stigma drivers and facilitators, including but not limited to beliefs, fears, lack of awareness about the DRTB and stigma, inability to clinically manage the condition, negative attitudes, and institutionalized procedures or practices.
Context	Available data from countries identified in the World Health Organization’s list of high TB- and drug-resistant TB burden countries (Supplemental Appendix 1).

**Figure 1.**
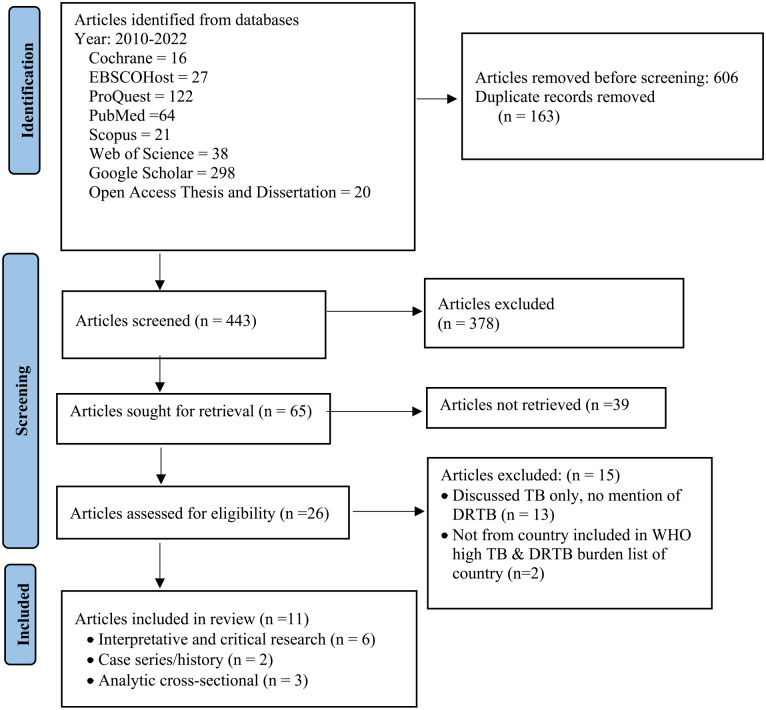
PRISMA-SCr flow diagram in examining the driver and facilitators of DRTB-related stigma among CHWs and recommendations to help address the stigma.

After de-duplication, the searches yielded 443 articles, 65 were selected for title and abstract evaluation, and 39 were excluded due to inclusion criteria ineligibility. After reviewing 26 full-text articles, 11 relevant articles were reviewed and synthesized. Figure 1 shows the PRISMA-ScR article flow from identification through inclusion.

### Inclusion Criteria

Eligibility of included studies were:

- focused on DRTB stigma among HCWs, or
- presented evidence that are associated to DRTB stigma among health workers, and
- participants from WHO high-TB/DRTB burden countries (Supplemental Appendix 2)

Studies pertaining to health professionals’ stigma toward TB, not DRTB, was excluded. DRTB stigma studies on patients and their families, and those from non-WHO high TB and DRTB-burden countries were excluded. Editorials and comments were excluded.

### Data Charting

Data charting was iterative to ensure comprehensive literature search. Reviewers independently and thoroughly evaluated the articles and charted the relevant material in a chart developed beforehand (Supplemental Appendix 3). Utilization of data charting was to prevent removing potential results of importance to this review’s synopsis.^35^ Endnote’s citations and full texts imported into NVivo were used to improve data charting and textual data source analysis. Citation specifics, study details, the verbatim accounts of stigma experience and the findings associated with stigma reduction were likewise charted.

The articles were examined using JBI’s quality assessment tool. Unlike a systematic review, which formally analyzes methodological quality for risk of bias, our scoping review broadly examined the quality of the studies to provide an overview of research activity on a topic.^36^ It was not intended to rank the articles based on the evidence provided, and no articles were excluded from the review based on their quality. Supplemental Appendix 4 contains a quality assessment of each study design included in this review.

## Results

### Included Studies’ Characteristics

This review comprised case series/case history (n = 2), analytical cross-sectional (n = 3), and interpretative and critical research (n-= 6), with most studies from South Africa (n = 8) and one each from Indonesia, the Philippines, and Tanzania. Nine studies used interviews and surveys, 3 used historical data, and no systematic reviews. Only 2 of 11 studies focused solely on DRTB stigma among HCWs. The other 9 studies focused on HCWs’ perspectives on DRTB care (n = 4), HCWs and DRTB infection control (IC) (n = 2), and DRTB infection among HCWs (n = 3). However, the studies found evidence of DRTB-related stigma among HCWs. Eight studies included a mix of health workers, while 3 studies solely included physicians. No studies solely included nurses, medical laboratory technologists, pharmacists, and other allied health workers. Supplemental Appendix 5 describes the included research by design. Table 2 summarizes the stigma drivers and facilitators, including verbatim quotes from the included studies.

**Table 2.** Summary of Included Studies in Examining the Evidence of DRTB Related Stigma Among HCWs in High TB and DRTB Countries.

Citation	Study design	Data collection	Target population	Study setting or country	Drivers of stigma	Facilitator of stigma	Verbatim quote descriptions of stigma experience	Verbatim quote recommendation for stigma reduction
Padayatchi et al.^40^	Case Series	Historical data	Physician	South Africa	Fear of contracting the disease; Stress in providing care; Shame; Feeling unsafe	Lack of infection control; Weak psychosocial support	*“Doctors felt the personal and professional stresses of carrying on work after being diagnosed with DRTB”* ^37^ *“Several doctors experienced difficulty and awkwardness re-integrating into a teaching environment where their illness have been publicised.”* ^37^ *“Doctors expressed feeling ashamed and blamed for acquiring TB as they were professionals who are expected to be fully aware of risk exposure yet ill-equipped to apply this knowledge to protect themselves.”* ^37^	*“Prioritization of infection control education and practice;* *Enhanced infection control education in medical curricula and practice, towards mitigating occupational risks;* *Adherence to legislation for occupational safety.”* ^37^ *“Psychological support and professional counselling should be a routine part of conventional medical care for doctors with TB in high-risk settings.”* ^37^
Kanjee et al.^39^	Cross-sectional Study	Questionnaire	HCWs	South Africa	Fear of contracting the disease; Feeling unsupported; Feeling isolated; Shame	Lack of infection control	*“As several staff members had died from confirmed TB/MDR-TB/XDR-TB, 82.1% and 42.9% of respondents were less willing to work in high-risk areas of the hospital or to work as an* HCW, *respectively.”*^38^ *“HCWs do not trust the health services to take care of them if they have TB”*^38^ *“HCWs fear potential rejection and stigma from staff if they have TB; and think it is shameful to admit they might have TB”*^38^	* “Facilities must implement multi-faceted TB IC facility and behavioural change interventions”* ^38^ *“Facilities should ensure confidentiality of staff health information and reduce the stigma in order to improve HCW uptake of personal diagnosis and other risk-reduction strategies.”* ^38^
Naidoo et al.^46^	Cross-sectional study	Self-administered questionnaire	Physicians (inlcuding 4 respondents with DRTB)	Sub-sahara Africa	Feeling discriminated, unsupported.	Lack of infection control; Lack of psychosocial support	*“It hurts when our own well-being is jeopardized, and our own colleagues and management show an uncaring attitude towards us.”* ^39^ *“Physicians regretted choosing clinical medicine as a career option, another stated being treated inappropriate by colleagues and criticised for taking sick leave”* ^39^	The study emphasizes the need for improved educational and awareness programs for all healthcare personnel, including hospital administrators, and changes in attitudes on the part of senior medical colleagues and administrators towards medical doctors with TB.
Tudor et al.^22^	Cross-sectional Study	Questionnaire	HCWs	South Africa	Fear of contracting the disease; Feeling isolated; Feeling discriminated; Feeling unsupported	Lack of infection control; Inequity in income protection	*“they were concerned about being isolated from their family for long periods of time “to be isolated and away from family and friends”* ^22^ *“how will society accept you, how will your colleagues treat you?”* ^22^ *“. . . getting infected with MDR- or XDR-TB and no one cares about what happens to me”* ^22^ *“HCWs do not receive danger allowance for working in MDR-/XDR-TB wards and the lack of compensation they would receive if they were to acquire MDR-/XDRTB”* ^22^ *“HCWs responded that they were concerned about stigma and the perceived lack of psychosocial support if they become ill.”* ^22^	*“It is imperative that efforts are made to improve IC and ensure safe working conditions for all HCWs.”*^22^ This study highlights training of HCWs in IC measures, and specifically on protecting self and others from the TB(DRTB) Recommended all HCWs, to have free access occupational health services and income protection.
Daftary and Padayatchi^42^	Qualitative Study	Interviews	HCWs	South Africa	Stress in providing care; Fear of contracting the disease	Lack of infection control	*“Junior HCWs expressed concern on nosocomial exposure due to patients no closing their mouths when coughing.”* ^40^	This study recommended addressing suboptimal infection control practices and complacency in the workplace through leadership and governance
Jaramillo et al.^45^	Qualitative Study	Interviews	HCWs	Philippines	Fear of contracting the disease Stress in providing DRTB care	Lack of infection control	*“There is poor infection control practices, fear, and limited capacity in rural health centers”* ^41^	This study recommended that professional development opportunities, oversight of personnel, and information campaigns should be in place to curb stigma in the community
Lyakurwa et al.^41^	Qualitative Study	Interview and focus group discussions	HCWs	Tanzania	Fear of contracting the disease. Feeling unsupported	Lack of knowledge in DRTB care, Lack of financial support	*“HCWs lacked confidence in the quality of care at their facility and fear DRTB because they have no experience in treating DRTB patients and assume having a higher risk to be infected by them”* ^42^ *“. . .no extra-duty pay for staff working on holidays”* ^42^	*“The teams proposed to train more HCWs, including facility managers in the training and ongoing mentorship”* ^42^
Probandari et al.^37^	Qualitative Study	Questionnaire	Primary Health Care health staff	Indonesia	Fear of contracting the disease; Feeling unsafe; Stress in providing care	Lack of infection control	*“Staff fear of being infected and feeling afraid to talk to MDRTB patients; they are stressed and fearful of conducting MDRTB care”* ^43^	The study emphasized the need for knowledge and capacity building for infection control The Study identified that training alone is not sufficient; and research is needed to evidence effective strategies that reduce stigma among health staff providing MDRTB care
Vanleeuw et al.^43^	Qualitative Study	Interviews	HCWs	South Africa	Fear of contracting the disease; Stress in providing care	Lack knowledge in DRTB program implementation; Lack of administrative support in addressing changes in DRTB care.	*“These doctors are scared of TB patients and refer them quickly.”* ^44^ *“Majority of HCWs at primary healthcare level did not have this experience and expressed concern with the sudden addition of DR-TB patients to their daily routine”* ^44^	The study recommended that attention is required as to how the decentralized DR-TB unit can be supported by district management and other healthcare providers.
Zelnick et al.^38^	Qualitative Study	Key informant interview and survey questionnaire	HCWs	South Africa	Fear of contracting the disease; Feeling unsafe; Feeling discriminated	Lack of infection control; Inequity in income protection	*“Nurse: TB is all over, so we* can’t *get a risk allowance. . .the only compensation we can get is being treated free when you are diagnosed with TB.”*^25^	This study recommended for evaluation of IC efforts and the decentralized management of DRTB as part of the initiatives to control DRTB and create as safe workplace.
Von Delft et al.^44^	Text and opinion	Historical data	Physicians	South Africa	Feeling isolated; Fear of discriminated; Fear of infection	Lack of infection control	*“The medical student with MDR tuberculosis experienced extreme social isolation, driven by a lack of understanding from family members and peers as well as the medical school administrator.”* ^45^ *“HCWs in low-resource settings with possible tuberculosis symptoms are already wary of presenting for testing and treatment, because of stigma and career implications; and fear of infecting family members.”* ^45^	This study recommends for an urgent action to improve IC implementation.

#### Stigma drivers

All studies identified more than one stigma driver, with an emphasis on fear. HCWs working in DRTB wards are most afraid about catching the disease, which could spread to their families and others.^22,37-45^ HCWs’ nosocomial infection concerns vary, reflecting workplace behaviors,^38,39,42,43^ and lack of knowledge on IC guidelines.^37,38,41,42^

Another stigma driver noted is stress from treating DRTB patients. Four studies found that HCWs were stressed about providing DRTB care, mostly due to a lack of experience,^37,40,42,43^ and confidence in their facility’s capability to provide DRTB care.^41^

Two studies mentioned HCWs’ stigma being perpetuated by their colleagues and peers. Example, HCWs were concerned about potential rejection or compromising work relationships if they became ill, whereas those who became ill faced discrimination and career implications.^39,44^ In 4 studies, infected HCWs’ psychological experiences were described as extensive, including depression, anxiety, resentment, paranoia, and intense fear of relapse or re-exposure.^22,40,44,46^ Two studies mentioned the fear of isolation or separation from family and friends.^22,46^

#### Stigma facilitators

Stigma facilitators are perceived influences that reduce or enhance stigmatizing behaviors.^47^ In healthcare, cultural norms, workplace safety requirements, and health policy are examples of stigma facilitators.^25^ Poor occupational safety standards, including a lack of IC, were most frequently regarded as a stigmatizing facilitator in the included studies. Seven South African studies reported HCWs’ worry with workplace poor IC practices and complacency^22,37-40,42,44^ with some HCWs not following IC advice.^39^ Six studies mentioned inadequate IC due to disparities in risk perceptions among co-workers, varying policy interpretations, or lack of knowledge about IC.^22,37,38,42,44,45^

Weak workplace structure support was also mentioned. Six studies identified HCWs’ low morale as a result of disparities in hazard compensation, job instability, and organizational bureaucracy.^22,37,38,40,44,45^ Also with “freeze hire policy” in place, HCWs felt helpless to provide support to colleagues, while others felt anxious about not receiving the necessary support to conduct the DRTB care program.^43^

#### Stigma reduction recommendations

Three key recommendations surfaced from the studies: (a) address IC issues; (b) increase the competence of healthcare workers; and (c) provide psychosocial assistance. In tackling IC, the recommendations centered on behavioral modification, skill enhancement, and implementation governance. One recommendation mentioned ensuring adequate IC supply,^39^ while others recommended educating HCWs in IC control^22,37^ and ensuring compliance with IC policy.^40^ Two studies recommend assessing the IC program and leadership to address HCWs’ mistrust in IC initiatives, traditional workplace practises, and workplace hierarchies that result in poor IC practices.^38,42^

Stigma reduction recommendations also included knowledge and capacity training, notably in the new DRTB care paradigm that includes primary health services. The recommendations addressed HCWs’ worry and anxiety around DRTB care changes by engaging and supporting them. One recommendation was for DRTB program managers to be mindful of the assistance required by the decentralized DRTB units.^43^ Three studies recommended training^41,43,45^; however, continued mentoring,^41^ and intensified information campaigns to curb DRTB stigma among HCWs and community were emphasized.^45^

Most agreed that HCWs’ safety and security during DRTB activities is a priority.^22,37,38,40,44,48^ For example, one study recommended ensuring the confidentiality of information in DRTB facilities to encourage testing and diagnosis among HCWs,^39^ while another recommended providing equitable compensation and free access to appropriate health services.^22^ But for Probandari et al,^37^ more research is needed to collect evidence about the effective strategies to reduce stigma among CHWs providing MDR-TB care.

## Discussions

We found few studies, predominantly from South Africa and one each from Indonesia, the Philippines, and Tanzania. No literature review DRTB stigma among HCWs was found, so far. The stigma associated with communicable diseases^49^ is consistent with this review. In this review, DRTB stigma is driven by fear of the disease, comparable to studies where HCWs reported stigma from infectious disease exposure.^50-52^ The inherent characteristics of DRTB, treatment toxicity, and poor treatment outcomes all contribute to stigmatization.^9,53^ We found that impacted HCWs engage in stigmatizing behaviors such as avoiding delivery care, transferring patients with DRTB quickly, or performing unnecessary overprotection IC practices.^37,43^ More concerning is the report that 82.1% of hospital staff and 42.9% of HCWs are less willing to work in DRTB-specific areas or continue to work as health workers.^39^

Our analysis found multiple stigma facilitators, with healthcare system social and structural variables having the biggest impact. IC was frequently mentioned as requiring proper execution, not adhering to, or having its policy interpreted differently among stigma facilitators. HCWs worried about inadequate IC practice and complacency in their health facilities.^22,[Bibr bibr38-00469580231180754]38-40,42,44,46^ Due to limited IC supply at health institutions, some HCWs were stigmatized for not following IC procedure.^39,46^ Even more alarming is the reluctance of other HCWs to adhere to the IC’s TB recommendations.^39^ We found that the IC reluctance was due to lack of knowledge regarding DRTB risks or conflicting interpretations of IC policy.^22,37,39,40,42,46^ Unsurprisingly, inadequate IC in the workplace facilitates HCWs’ fear of the disease.

Working with stigmatized individuals or diseases promotes stigmatization^54,55^; and this is echoed in this review. We identified the stigmatizing experiences of HCWs, such as discrimination, isolation, rejection, and shame. For example, co-workers may reject HCWs if they become ill, but those who contracted DRTB delayed testing and diagnosis out of fear of discrimination and career implications.^39^ Some HCWs with DRTB suffered anxiety and depression as a result of isolation from family and friends, or lost contact with colleagues due to extended absences from work.^22,40,44,46^ Yet concerning is leaving their families, particularly their children, to pursue DRTB treatment.^22^

We, likewise, identified discriminating behaviors in the workplace. For instance, senior HCWs do not feel themselves to be in danger from the disease, hence blaming junior colleagues for IC wastage and limiting their access to supplies.^42^ Arguably, stigmatizing tendencies are prevalent among HCWs who lack knowledge and training in DRTB.^41,45,48^ We also identified policies characterized by disparities in financial security among HCWs. Compared to those working in other high-risk settings, like HIV, some HCWs in DRTB wards do not receive a “danger pays,” day off on holidays, or compensation if they acquire DRTB.^22,41^ In another instance, some HCWs were excluded from training opportunities, thus felt discriminated.^43,45^

Additionally, we found that decentralization of DRTB care to community health centers also stressed HCWs. Fear and anxiety resulted from the increase in workload, with HCWs expressing concern about the lack of assistance from coordinators to guide them through the adjustments and aid them with patient-related issues, and coordinators experiencing stress due to excessive workloads.^41-43^ We found that an appointment moratorium prevented DRTB supervisory assistance.^43^ Our findings support WHO’s^56^ assertion that a lack of appropriate structures and support at work could adversely impact HCWs’ mental health, thereby hindering their capacity to enjoy and perform well at work.

Of note, data on the causes and facilitators of stigma are necessary for determining the most effective stigma reduction intervention in a given environment.^25^ In this review, we identified the stigma drivers and facilitators that could assist the DRTB community with identifying the policies that need to be modified to promote a stigma-free healthcare environment. With our findings, we urge DRTB implementers and policymakers to enable policy revisions, notably regarding occupational health and safety, and HCW income protection.

Most studies concluded with stigma reduction interventions, emphasizing DRTB knowledge, current training (including refresher training), and capacity building on IC, and enhancing administrative IC operational standards. However, HCWs were concerned that training could not overcome stigma; implying that IC training could help reduce barriers to implementing IC, but not fear. This finding supports the claim that training alone is insufficient for sustainability if the healthcare system lacks infrastructure.^57^ We found that continual mentoring and supportive supervision that improves knowledge and abilities may reduce stigma better because additional attention and on-the-job mentoring were deemed essential for HCWs to acquire confidence in providing DRTB care.^41^

Notably, stigmatization in DRTB workforce is viewed as a result of both individual and institutional factors. This review, however, falls short of identifying stigma drivers and facilitators from multiple countries due to a significant lack of literature that explicitly characterizes the domains in DRTB-related stigma. The WHO^58^ identifies many countries with significant TB and DRTB burdens combined; yet, only few countries have investigated the stigmatization of HCWs providing DRTB care. Also, workplace DRTB-associated stigma has just recently been recognized. We urge stakeholders, including academics and policymakers, to investigate stigma routes at facility level, focusing on HCWs experiencing stigma and those perpetuating stigma. Similarly, it is important to identify and understand the elements that facilitate and mediate stigmatization in a multi-level context, addressing the ecological and social pathways to DRTB stigma.^25^

### Strengths and Limitations

This review summarizes stigma among HCWs caring DRTB patients from TB- and DRTB-affected countries. So far, this is the first review to focus solely on DRTB stigma and HCWs. With limited studies found, our review is unable to comprehensively picture DRTB-associated stigma among HCWs; a significant information gap requiring attention. HCWs’ DRTB stigma needs further study at the facility, program, and national levels.

This review has some limitations. Despite attempts to be as exhaustive as possible, this review may not have found every study in the published and gray literature. The literature search’s inclusivity in terms of period, language, and study location may have missed some relevant studies. Our review is focused on studies conducted in countries with the highest TB and DRTB burdens, according to the WHO. While many TB- and DRTB-burden nations have non-English-speaking populations, this review may have omitted non-English studies. The MeSH search included phrases related to DRTB and health professionals, but they may not have been in the title or abstract.

Our review objective is to provide breadth rather than depth of evidence regarding DRTB related stigma confronting HCWs; thus, included studies regardless of research design, or outcome. Studies’ quality gives an overview of research effort on a topic but is not a reason for exclusion. The studies’ variabilities limit the conduct of meta-analysis, which is an inherent limitation of scoping reviews. We ensured a complete and transparent presentation of results by utilizing the recommended conventions of PRISMA-Scr.

### Research and Practice Implications

Healthcare professionals and DRTB stigma have not yet been extensively studied. More research is needed to fully reflect DRTB stigma in healthcare and develop the requisite stigma reduction strategy and evaluate its efficacy. To overcome inequities and create effective, viable solutions that discourage stigma facilitators, policymakers must reconsider TB and DRTB policies, notably IC and risk-payments. Researchers need more data to critically identify the factors that contribute to and facilitate stigmatization among HCWs. It is prudent to take note of the WHO’s recommendation to expand country-specific DRTB research and Stangl et al’s^25^ recommendation to investigate stigmatization across its socio-ecological range.

## Conclusions

Our scoping research aims to identify the drivers and facilitators of stigma around HCWs providing crucial services for DRTB, particularly in countries with a high prevalence of TB and DRTB, as well as recommendations to reduce stigma. Despite the low number of studies reviewed, the findings imply that the stigma associated with DRTB in the healthcare sector is multifaceted and largely driven by the fear of infection. Other collective stigmas experienced by HCWs included discrimination, loneliness, insecurity, embarrassment and stress. Inconsistencies health workplace structure, such as conflicting IC interpretations and culture and values, also significantly contribute to the stigmatization among HCWs. Making HCWs feel safe while conducting DRTB activities is a priority issue that should be addressed. There are gaps in the literature, with no studies were found in most of the WHO’s high-burden TB and DRTB nations. Further research regarding country-specific and multi-level DRTB-related stigma among HCWs is necessary.

## Supplemental Material

sj-docx-1-inq-10.1177_00469580231180754 – Supplemental material for Drug-Resistant Tuberculosis Stigma Among HealthCare Workers Toward the Development of a Stigma-Reduction Strategy: A Scoping Review Click here for additional data file. Supplemental material, sj-docx-1-inq-10.1177_00469580231180754 for Drug-Resistant Tuberculosis Stigma Among HealthCare Workers Toward the Development of a Stigma-Reduction Strategy: A Scoping Review by Lolita Liboon Aranas, Khorshed Alam, Prajwal Gyawali and Rashidul Mahumud Alam in INQUIRY: The Journal of Health Care Organization, Provision, and Financing
