# Supplementary material for: Drug-Resistant Tuberculosis Stigma Among HealthCare Workers Toward the Development of a Stigma-Reduction Strategy: A Scoping Review
Source: Inquiry. 2023 Jun 13;60:00469580231180754. doi: 10.1177/00469580231180754 (PMC10286532; doi:10.1177/00469580231180754)
Supplement: sj-docx-1-inq-10.1177_00469580231180754 – Supplemental material for Drug-Resistant Tuberculosis Stigma Among HealthCare Workers Toward the Development of a Stigma-Reduction Strategy: A Scoping Review [file sj-docx-1-inq-10.1177_00469580231180754.docx]

| The search strategy was designed around the aims of the review and included two key concepts, DRTB and stigma. The search was designed to align with the 3-step strategy recommended by JBI scoping review guidelines. First was the pilot search of the PubMed and EBSCO databases using Medical Subject Heading (MeSH) terms to identify the articles on the topic. Second step was the creation of the search protocol using the identified text terms in the titles, abstracts, and keywords and used the protocol to in the full search of databases CINAHL, Cochrane, ProQuest, Scopus, Web of Science. The systematic search utilized the terms “drug-resistant tuberculosis”, “drug-resistant TB”, “multidrug-resistant tuberculosis”, “multidrug-resistant TB”, “stigma”, “stigmatization”, “stigmatize”, “prejudice”, “attitude, “discrimination”, “health workers”, “medical workers”, “healthcare professionals”; and Boolean terms (AND/OR) as separators. Last was the search for gray literature and additional resources in electronic sources such as Google Scholar, ProQuest Dissertation, Open Access Thesis and Dissertations and researchgate. The search filters applied in English from 2010 to 2022 to identify the more recent concern regarding DRTB stigma among health workers. The bibliographies of the included articles were scanned to identify additional relevant articles.   \| Appendix 1. Preliminary Data Base Search Strategies File_Pilot \| \| \| --- \| --- \| \| 1. **Insert name of database**:   **Search conducted on**:  **Interface**  **Search Screen**  **Database**  **Search term**  **Limiters**  **Expanders**  **Search modes**  **Number of records retrieved by the search** \| EBSCOhost  22 September 2022  EBSCOhost Research Databases  Advanced Search  Academic Search Ultimate  drug-resistant tuberculosis OR drug resistant tb OR multidrug-resistant tuberculosis OR multidrug-resistant TB AND (stigma or prejudice or attitude or discrimination) OR (stigmatization or stigmatisation) AND healthcare professionals OR healthcare workers OR ( medical workers or medical staff or health care worker or healthcare employees )  Published Date: 20100101-20220931; Document Type: Abstract; Language: English  Apply equivalent subjects  Boolean/Phrase  379 articles \| \| 1. **Insert name of database**   **Search conducted on**  **Interface**  **Search Screen**  **Search term**  **Expanders**  **Search modes**  **Number of records retrieved by the search** \| Scopus  22 September 2022  <https://www-scopus-com.ezproxy.usq.edu.au/search/form>.  Advanced Search  ( TITLE-ABS-KEY ( drug-resistant AND tuberculosis ) OR TITLE-ABS-KEY ( drug-resistant AND tb ) OR TITLE-ABS-KEY ( multidrug-resistant AND tuberculosis ) OR TITLE-ABS-KEY ( multidrug-resistant AND tb ) AND TITLE-ABS-KEY ( stigma ) OR TITLE-ABS-KEY ( stigmatisation ) OR TITLE-ABS-KEY ( stigmatization ) OR TITLE-ABS-KEY ( stigmatise ) OR TITLE-ABS-KEY ( prejudice ) OR TITLE-ABS-KEY ( attitude ) OR TITLE-ABS-KEY ( discrimination ) AND TITLE-ABS-KEY ( health AND workers ) OR TITLE-ABS-KEY ( health AND professionals ) OR TITLE-ABS-KEY ( medical AND workers ) OR TITLE-ABS-KEY ( medical AND professional ) ) AND PUBYEAR > 2009  Apply equivalent subjects  Boolean/Phrase  108 articles \| |
| --- | --- | --- | --- | --- | --- | --- |

## Appendix 1. Search strategy for examining DRTB stigma among healthcare workers

| **Appendix 2: World Health Organization list of high TB- and high DRTB-burden countries** | | |
| --- | --- | --- |
| **30 High TB burden countries** | |  |
| Angola | Philippines |  |
| Bangladesh | Russia |  |
| Brazil | South Africa |  |
| China | Thailand |  |
| DPR Korea | UR Tanzania |  |
| DR Congo | Vietnam |  |
| Ethiopia | Cambodia |  |
| India | Central African Republic |  |
| Indonesia | Congo |  |
| Kenya | Lesotho |  |
| Mozambique | Liberia |  |
| Myanmar | Namibia |  |
| Nigeria | Papua New Guinea |  |
| Pakistan | Sierra Leone |  |
| Zimbabwe | Zambia |  |
| **30 high drug-resistant TB countries** | |  |
| Bangaladesh | Philippines |  |
| China | Russia |  |
| DPR Korea | South Africa |  |
| DR Congo | Thailand |  |
| Ethiopia | Ukraine |  |
| India | Uzbekistan |  |
| Indonesia | Vietnam |  |
| Kazakhstan | Angola |  |
| Kenya | Azerbaijan |  |
| Mozambique | Belarus |  |
| Myanmar | Krygyzstan |  |
| Nigeria | Papua New Guinea |  |
| Pakistan | Peru |  |
| Reprubic of Moldova | Somalia |  |
| Zimbabwe | Tajikistan |  |

| **Appendix 3: Data extraction chart for the scoping review in mapping the evidence of drug-resistant tuberculosis related stigma among health workers in high TB- and drug-resistant-TB burden countries** | | |
| --- | --- | --- |
| Key Domain | Sub-domain | Description |
| Citation details | Name of Authors | Indicate the name of the authors |
|  | Year of Publication | Indicate the year when the article was published |
|  | Title of Publication | Indicate the full title of the article |
|  | DOI | Indicate, if provided |
| Language |  | Only English language |
| Type of publication |  | Identifies the article if original review, or grey |
| Source Title |  | Identifies the name of the journal |
| Study details | Aims/Objectives | Describe the aims and objected stated in the study |
|  | Study location | Indicate the study sites (country or region) |
|  | Sample characteristics | Indicate the group of health workers involved in the study |
|  | Sample size | Indicate the number of participants in the study |
|  | Study design | Indicate the methodology adopted e.g. qualitative, quantitative, mixed-method, case study, review. |
|  | Data collection | Indicate the type of data (primary or secondary) and how the data was collected (e.g. interview, survey, etc.) |
|  | Key themes or type of stigma assessed | Indicate the stigma domain (e.g. fear), if primarily identified in the title or objectives of the study |
|  | Description of stigma association | Describe the association of stigma in the finding of the study |
|  | Significant mediators/moderators | Indicate of interventions were identified in the study |
|  | Key findings | Describe the main results (evidence, concept, themes) and link to the scoping review question and objectives |
|  | Study limitations | Indicate the limitations of the study |
| Abstract |  | A copy of the study abstract |

## **Appendix 4. Quality Assessment of the Studies**

### Appendix 4A: Case-series/history

| Question | Citation | | |
| --- | --- | --- | --- |
|  | Padayatchi et al., 2010 | Von Delft et al., 2016 |  |
| Were there clear criteria for inclusion in the case series? | Y | Y |  |
| Was the condition measured in a standard, reliable way for all participants included in the case series? | Y | Not Clear |  |
| Were valid methods used for identification of the condition for all participants included in the case series? | Y | Not clear |  |
| Did the case series have consecutive inclusion of participants? | Y | No |  |
| Did the case series have complete inclusion of participants? | Y | Yes |  |
| Was there clear reporting of the demographics of the participants in the study? | Y | No |  |
| Was there clear reporting of clinical information of the participants? | Y | Yes |  |
| Were the outcomes or follow up results of cases clearly reported? | Y | No |  |
| Was there clear reporting of the presenting site(s)/clinic(s) demographic information? | Y | No Clear |  |
| Was statistical analysis appropriate? | y | No |  |

### Appendix 4B: Analytical cross-sectional

| Question | Citation | | |
| --- | --- | --- | --- |
|  | Kanjee et al., 2011 | Tudor et al., 2013 | Naidoo et al., 2013 |
| Were the criteria for inclusion in the sample clearly defined? | Y | Y | Y |
| Were the study subjects and the setting described in detail? | Y | Y | Y |
| Was the exposure measured in a valid and reliable way? | Y | Y | Y |
| Were objective, standard criteria used for measurement of the condition? | Y | Y | Y |
| Were confounding factors identified? | Y | Y | Y |
| Were strategies to deal with confounding factors stated? | Y | Y | Y |
| Were the outcomes measured in a valid and reliable way? | Y | Y | Y |
| Was appropriate statistical analysis used? | Y | Y | Y |

### Appendix 4C: Interpretative and Critical Research

| Question | Citation | | | | | |
| --- | --- | --- | --- | --- | --- | --- |
|  | Daftary A, Padayatchi N. 2016. | Lyakurwa et al, 2021 | Probandari et al., 2019 | Vanleeuw et al**.,** 2020 | Zelnick et al., 2013 | Jaramillo et al., 2022 |
| Is there congruity between the stated philosophical perspective and the research methodology? | Y | Y | Y | Y | Y | Y |
| Is there congruity between the research methodology and the research question or objectives? | Y | Y | Y | Y | Y | Y |
| Is there congruity between the research methodology and the methods used to collect data? | Y | Y | Y | Y | Y | Y |
| Is there congruity between the research methodology and the representation and analysis of data? | Y | Y | Y | Y | Y | Y |
| Is there congruity between the research methodology and the interpretation of results? | Y | Y | Y | Y | Y | Y |
| Is there a statement locating the researcher culturally or theoretically? | Y | Y | Y | Y | Y | Y |
| Is the influence of the researcher on the research, and vice-versa, addressed? | Y | Y | Y | Y | Y | Y |
| Are participants, and their voices, adequately represented? | Y | Y | Y | Y | Y | Y |
| Is the research ethical according to current criteria or, for recent studies, and is there evidence of ethical approval by an appropriate body? | Y | N | Y | Y | Y | N |
| Do the conclusions drawn in the research report flow from the analysis, or interpretation, of the data? | Y | Y | Y | Y | Y | Y |

**Appendix 5: Characteristics of Included Studies**

# Appendix 5A: Characteristics of Included Studies - Interpretive and Critical Research Form

| **Study** | **Methods for data collection and analysis** | **Country** | **Phenomena of interest** | **Setting/ context/ culture** | **Participant characteristics and sample size** | **Description of main results** |
| --- | --- | --- | --- | --- | --- | --- |
| Lyakurwa D, Lyimo J, Mulder C, Pelzer PT, Koppelaar I, Heus M. 2021. | Interview and focus group discussions analysed using thematic analysis | Tanzania | Drug-resistant TB care performance among HCWs | Drug-resistant TB facilities | 454 HCWs | HCWs developed DRTB competencies after training and mentoring. HCWs main challenges un DRTB care were delays in laboratory results, stigma and workforce shortage. |
| Vanleeuw L, Atkins S, Zembe-Mkabile W, Loveday M. 2020. | Interview anlayzed thematically | South Africa | Perceptions and experiences of HCWs in DRTB care | Primary health care facilities and DRTB hospitals | HCWs | The introduction of DRTB care in primary care facilities created fear and anxiety among HCWs. HCWs felt unsupported and expressed feeling isolated which they feel impacts on the quality of care they provide to their patients. |
| Zelnick JR, Gibbs A, Loveday M, Padayatchi N, O’Donnell MR. 2013. | Key informant interview and survey questionnaire analysed using deductive analysis | South Africa | Workplace exposure to DRTB | Hospitals | 55 HCWs | The key themes identified in the study were lack of infection control measures, stigma and support in workplace. |
| Jaramillo J. 2022. | Interview analysed using framework analysis | Philippines | Barriers associated with DRTB care service delivery | DRTB treatment facilities | 272 HCWs, mainly nurses and physicians | Results identified five themes included: nurses not feeling empowered; poor infection control practices, fear and limited capacity in rural health centres, limited government support mechanisms on DRTB elimination activities. |
| Daftary A, Padayatchi N. 2016. | Interviews | South Africa | HCW experiences with service delivery for multi-drug resistant and extensively drug-resistant tuberculosis (MDR/XDR-TB and TB-HIV | Centralised tertiary TB site | 17 health care workers (primary care nurses and doctors | A thematic result identified included 1) weak of personal infection control practices among HCWs due to work culture characterised by low motivation, disparate risk perceptions and practices, physical discomfort and problems managing patients, 2) stigma associated with MDR/XDRTB is worst that HIV and maybe perpetuated by those less familiar with the disease, 3) administration restrictions, workplace norms and provider mindsets imped effective integration of HIV and MDR/XDR-TB services, 4) HCWs who struggle with daily routines of MDR/XDR-TB treatment supervision are increasingly supportive of treatment literacy and self-administration |
| Probandari A, Sanjoto H, Mahanani MR, Azizatunnisa L, Widayati S. 2019. | Structured questionnaire, in-depth interviews and observations analyzed using content analysis technique | Indonesia | Issues of safety and stigma among health staff | Primary health care facilities | 123 health staff, 17 primary health care facilities | The knowledge and motivation to follow Multidrug-resistant TB care protocols are suboptimal. HCWs feeling unsafe is related to stigmatising attitudes in providing MDRTB care. |

# Appendix 5B: Characteristics of Included Studies - Case Series/History Form

| **Study** | **Country** | **Setting/context** | **Participant characteristics** | **Groups** | **Outcomes measured** | **Description of main results** |
| --- | --- | --- | --- | --- | --- | --- |
| Padayatchi N, Daftary A, Moodley T, Madansein R, Ramjee A. 2010. | South Africa | Dry-resistant TB hospital | HCWs | 5 doctors with primary DRTB | Psychological impacts of DRTB illness among doctors | Content analysis revealed five themes prolonged morbidity, psychological impact, poor infection control , weak support structures and attrition from the field. |
| Von Delft A, Dramowski A, Sifumba Z, Mosidi T, Ting TX, Von Delft D, et al. 2016. | South Africa | DRTB facilities in low-resource settings | Healthcare workers | 3 physicians working in DRTB facilities | Physical and psychological impact of DRTB on the three physicians who got ill of the disease | The isolation and fear among healthcare workers was extreme. As they have seen their coworkers pass away from the illness, they are concerned for their safety. |

# Appendix 5C: Characteristics of Included Studies - Analytical Cross-Sectional Study Form

| **Study** | **Country** | **Setting/context** | **Participant characteristics** | **Groups** | **Outcomes measured** | **Main description of results** |
| --- | --- | --- | --- | --- | --- | --- |
| Tudor C, Mphahlele M, Van der Walt M, Farley JE. 2013. | South Africa | Drug-resistant TB hospitals across South Africa | HCWs | 286 nurses38 medical officers10 others | Personal concerns about HCWs fears of contracting MDR-/XDR-TB | Thematic analysis identified fears associated with the personal risks of acquiring drug-resistant TB, treatment course, financial implications, family concerns, working environment and psychosocial issues. |
| Naidoo A, Naidoo SS, Gathiram P, Lalloo UG. 2013. | South Africa | Public and private sector clinics and hospitals | HCWs | Female doctors (19)Male doctors (21) | Experiences, attitudes and perceptions of medical doctors following treatment for TB within the healthcare system. | Majority of the participants expressed concerns regarding lack of infection control at the workplace, delays in diagnosis, negative attitudes of senior colleagues and administrators. |
| Kanjee Z, Catterick K, Moll AP, Amico KR, Friedland GH. 2011. | South Africa | District hospital | HCWs (n=57) | 43 female HCWs14 male HCWs | Tuberculosis infection control knowledge, attitude and practice among hospital staff | Findings showed that staff concerns involved confidentiality of staff health information, stigma of TB and HIV, inadequate resources for infection control, and patient non-compliance. Many staff were less willing to continue as healthcare worker because of staff deaths caused by TB/MDR/XDR-TB. |

| Study: Tudor C, Mphahlele M, Van der Walt M, Farley JE. 2013. | |
| --- | --- |
| Finding | A major concern raised by HCWs was that they do not receive danger allowance for working in MDR-/XDR-TB wards and the lack of compensation they would receive if they were to acquire MDR-/XDRTB. - how the lack of compensation for illness may affect family. (C) |
| Illustration | Financial implications |
| Finding | A nurse stated, ‘. . . getting infected with MDR- or XDR-TB and no one cares about what happens to me’. (C) |
| Illustration | Feeling unsupported |
| Finding | "Fear of separation from family members if they needed to undergo treatment for MDR-/XDR-TB and the impact that illness would have on other family members were common concerns. Several mentioned that they were worried about dying of MDR-/XDRTB and leaving their children; Others mentioned that they were concerned about being isolated from their family for long periods of time ‘to be isolated and away from family and friends." (C) |
| Illustration | Fear of isolation |
| Finding | "Several HCWs responded that they were concerned about stigma and the perceived lack of psychosocial support if they become ill." (C) |
| Illustration | Lack of psychosocial support |
| Finding | "One nurse stated, ‘how will society accept you, how will your colleagues treat you?’ Another nurse responded, ‘. . . stigma and discrimination by colleagues." (C) |
| Illustration | Feeling discriminated |
| Finding | "Several HCWs responded that they were concerned about stigma and the perceived lack of psychosocial support if they become ill." (C) |
| Illustration | Feeling discriminated |
| Finding | "...if UVG are not working, I'm threatened because I'm at risk of getting MDR/XDR-TB..." (Nurse) (C) |
| Illustration | Lack of equipment for infection control |
| Finding | "...poor ventilation, working in busy and congregate settings, working with undiagnosed MDR/XDR patients". (medical officer) (C) |
| Illustration | Lack of infection control |
| Finding | "...this hospital we don not have green N95 mask. I cannot trust the N95 that we are wearing, and we do not have UV lights". (nurse) (C) |
| Illustration | Lack of resources for infection control |
| Study: Daftary and Padayatchi (2016) | |
| Finding | "Suboptimal infection control practices and complacency in the workplace must be addressed through leadership and governance." (C) |
| Illustration | Complacent infection control practices |
| Finding | Junior HCWs expressed concern on nosocomial exposure due to patients no closing their mouths when coughing (C) |
| Illustration | Nosocomial exposure |
| Study: Jaramillo et al. (2022) | |
| Finding | "some respondents mentioned working closely with rural health units to increase local staff knowledge about TB infection control and prevention measures." (C) |
| Illustration | Poor infection control |
| Finding | "...professional development afforded to TB-hired staff which are not extended to organic staff and impact morale" (C) |
| Illustration | Feeling discriminated |
| Finding | "...low observance of infection control guidelines." (C) |
| Illustration | Lack of infection control |
| Finding | "A misinformed healthcare worker is very tragic…The stigma comes from the barangay health workers or the informal health workers who are not trained properly.” (C) |
| Illustration | Misinformation |
| Study: Kanjee et al. (2011) | |
| Finding | "As several staff members had died from confirmed TB/MDR-TB/XDR-TB, 82.1% and 42.9% of respondents were less willing to work in high-risk areas of the hospital or to work as an HCW, respectively." (C) |
| Illustration | Unwillingness to work in DRTB ward |
| Finding | "...inconsistent cough hygiene among patients." (C) |
| Illustration | Fear of contracting the disease |
| Finding | ‘HCWs do not trust the health services to take care of them if they have TB.’ (C) |
| Illustration | Feeling unsupported |
| Finding | ‘HCWs fear potential rejection and stigma from staff if they have TB.' (C) |
| Illustration | Feeling unsupported |
| Finding | ‘HCWs think it is shameful to admit they might have TB.’ (C) |
| Illustration | Shame |
| Finding | "Facilities must enable implementation by providing an adequate supply of necessary resources, such as respirators and cough hygiene materials." (C) |
| Illustration | Lack of infection control |
| Finding | "Facilities should ensure confidentiality of staff health information and reduce the stigma of TB/HIV in order to improve HCW uptake of personal diagnosis and other risk-reduction strategies." (C) |
| Illustration | Concerns on confidentiality |
| Study: Lyakurwa et al. (2021) | |
| Finding | "HCWs lacked confidence in the quality of care at their facility and fear DRTB because they have no experience in treating DRTB patients and assume having a higher risk to be infected by them." (C) |
| Illustration | High risk of infection |
| Finding | "...trainers expressed their hesitations mainly due to lack of adequate facility equipment and infrastructure and insufficient infection prevention and control practices." (C) |
| Illustration | Inadequate infrastructure for infection control and |
| Study: Naidoo et al. (2013) | |
| Finding | "It hurts when our own well-being is jeopardized, and our own colleagues and management show an uncaring attitude towards us." (C) |
| Illustration | Feeling discriminated and unsupported |
| Finding | "Physicians regretted choosing clinical medicine as a career option, another stated being treated inappropriate by colleagues and criticised for taking sick leave; another felt angry and resentment towards TB patients for having infected them." (C) |
| Illustration | Feeling of anxiety |
| Finding | "The majority expressed concerns regarding lack of infection control at the workplace and delays in TB diagnosis." (C) |
| Illustration | Lack of infection control |
| Study: Padayatchi et al. (2010) | |
| Finding | "Doctors felt the personal and professional stresses of carrying on work after being diagnosed with DRTB." (C) |
| Illustration | Stress in providing care |
| Finding | "Several doctors experienced difficulty and awkwardness re-integrating into a teaching environment where their illness have been publicised." (C) |
| Illustration | Shame |
| Finding | "Doctors expressed feeling ashamed and blamed for acquiring TB as they were professionals who are expected to be fully aware of risk exposure yet ill-equipped to apply this knowledge to protect themselves. " (C) |
| Illustration | Shame |
| Finding | "...no specific strategy to mitigate airborne contagion." "Face masks were seldom available". (C) |
| Illustration | Lack of resources for infection control |
| Finding | "They were disenchanted by the bureaucratic difficulties faced with resuming work after having become patients themselves." (C) |
| Illustration | Weak support structure |
| Study: Probandari et al. (2019) | |
| Finding | "Staff fear of being infected and feeling afraid to talk to MDRTB patients." (C) |
| Illustration | Fear of contracting the disease |
| Finding | "Health staff stated about feeling stressed and fearful of conducting MDRTB care." (C) |
| Illustration | Stress in providing care |
| Finding | "...the study revealed that the knowledge of health staff about infection control protocols was inadequate." (C) |
| Illustration | Lack of knowledge in infection control |
| Finding | ...observation in 17 PHCs showed suboptimal infrastructure and fidelity of activities to infection control protocols." (C) |
| Illustration | Suboptimal infection control protocols |
| Study: Vanleeuw et al. (2020) | |
| Finding | "These doctors are scared of TB patients and refer them quickly." (C) |
| Illustration | Fear of contracting the disease |
| Finding | "Majority of HCWs at primary healthcare level did not have this experience and expressed concern with the sudden addition of DR-TB patients to their daily routine." (C) |
| Illustration | Stress in providing DRTB care |
| Study: Von Delft et al. (2016) | |
| Finding | "The medical student with MDR tuberculosis experienced extreme social isolation, driven by a lack of understanding from family members and peers as well as the medical school administrator." (C) |
| Illustration | Feeling isolated |
| Finding | "HCWs in low-resource settings with possible tuberculosis symptoms are already wary of presenting for testing and treatment, because of stigma and career implications." (C) |
| Illustration | Feeling discriminated |
| Finding | "Fear of infecting family members." (C) |
| Illustration | Fear of infection |
| Finding | "All HCWs, including volunteers and trainees, should have free access to appropriate occupational health services, income protection, and/or compensation in line with ILO workplace standards. Support broad social awareness campaigns that target stigma and discrimination by celebrating survivors and “normalizing” preventive and care-seeking behavior." (C) |
| Illustration | Lack of support in workplace |
| Study: Zelnick et al. (2013) | |
| Finding | "Patients are not diagnosed when they come in, so we are exposed." (C) |
| Illustration | Fear of contracting the disease |
| Finding | "TB is all over, so we can’t get a risk allowance…the only compensation we can get is being treated free when you are diagnosed with TB. HCWs expressed frustrations over not given danger pay." (C) |
| Illustration | Feeling discriminated |
| Finding | "Lack of resources and distrust of infection control efforts among HCWs." (C) |
| Illustration | Lack of infection control |
